# Is high exposure to antisocial media content associated with increased participation in malicious online trolling? exploring the moderated mediation model of hostile attribution bias and empathy

**DOI:** 10.1186/s40359-024-01898-0

**Published:** 2024-07-19

**Authors:** Yuedong Qiu, Qi Sun, Biyun Wu, Fang Li

**Affiliations:** 1 School of Psychology, Sichuan Normal University, Chengdu, China; 2 Center of Mental Health Education, Guangzhou University of Chinese Medicine, Guangzhou, China

**Keywords:** Malicious online trolling, Antisocial media exposure, Hostile attribution bias, Empathy

## Abstract

Malicious online trolling is prevalent among Chinese college students and has recently garnered extensive attention from researchers due to the substantial harm it causes to the victims and the damage it inflicts on the online environment. Most previous studies have focused on examining how personal traits related to malicious online trolling. Further comprehensive research is needed to explore the mechanisms linking external environmental factors (antisocial media exposure) and malicious online trolling. A total of 1259 Chinese college students completed questionnaires regarding malicious online trolling, antisocial media exposure, hostile attribution bias, and empathy. The results indicated a positive association between antisocial media exposure and malicious online trolling among Chinese college students, with hostile attribution bias serving as a mediating factor. Furthermore, the direct and mediated paths between antisocial media exposure and malicious online trolling were moderated by empathy. Specifically, as the level of empathy increased among college students, the relations between the variables all weakened. Excessive exposure to antisocial media content among college students may trigger hostile attribution bias and lead to more malicious online trolling behavior. However, the relation between antisocial media exposure and malicious online trolling, hostile attribution bias and malicious online trolling, was attenuated when college students’ empathy levels were high.

## Introduction

As network and communication technology has advanced, social networking platforms (such as TikTok, Weibo, etc.) have become essential communication channels for people to obtain information. By June 2023, China had a total of 1.079 billion Internet users [1]. indicating the significant influence of social media on contemporary social life. Users’ expressions on social networking platforms are not limited by time and space. The anonymity and low accountability of cyberspace make it easy for people to deviate from the principle of seeking truth from facts [2], leading to many problematic online behaviors [3, 4], such as online trolling. Online trolling involves deliberately posting provocative and inflammatory content on social networking platforms to trigger meaningless debates [5, 6]. Malicious online trolling is a complex phenomenon with various motivations, forms, and consequences [7], and malicious online trolling is a type of online trolling. As a typical offensive behavior, its content is generally offensive, deceptive and destructive [5]. Malicious trolls can usually be seen in places where personal opinions can be expressed on the Internet. They take pleasure in the pain of their victims [8]. Meaningless disruption of the online environment is an important feature that distinguishes malicious online trolling from antisocial behaviors such as cyberbullying [3]. This destruction not only harms the friendly atmosphere of online communication but also hinders the development of online platforms [9]. More seriously, malicious online trolling can cause various adverse effects on Internet users, including anxiety, depression, and other physical problems [10, 11], as well as increased self-harm and suicidal thoughts [12, 13]. Given the serious consequences that malicious online trolling might have led to, this study focused on exploring the predictive and preventive factors of malicious online trolling, which was of great significance for reducing malicious behavior in the online environment and protecting users’ mental health.

To date, there has been limited research on the factors and mechanisms influencing malicious online trolling, primarily focusing on individual factors such as dark personality traits, loneliness, and trait mindfulness. Few studies have investigated the antecedents and underlying mechanisms of malicious online trolling from the perspective of the online environment. In fact, with individuals’ increasing dependence on the Internet, the online environment has become an essential factor influencing individuals’ psychology and behavior [14]. Exposure to negative content in the online environment, such as false information, violent behavior, and hate messages, may heighten an individual’s inclination to engage in undesirable behaviors [15, 16]. Previous studies have established a connection between being exposed to harmful online content and engaging in risky behaviors among young people [17]. Another study has found a connection between the consumption of violent online content and individual cyberbullying [18]. In this study, antisocial media exposure (online environmental factors) was chosen to explore its relationship and mechanisms with malicious online trolling.

**Fig. 1:**
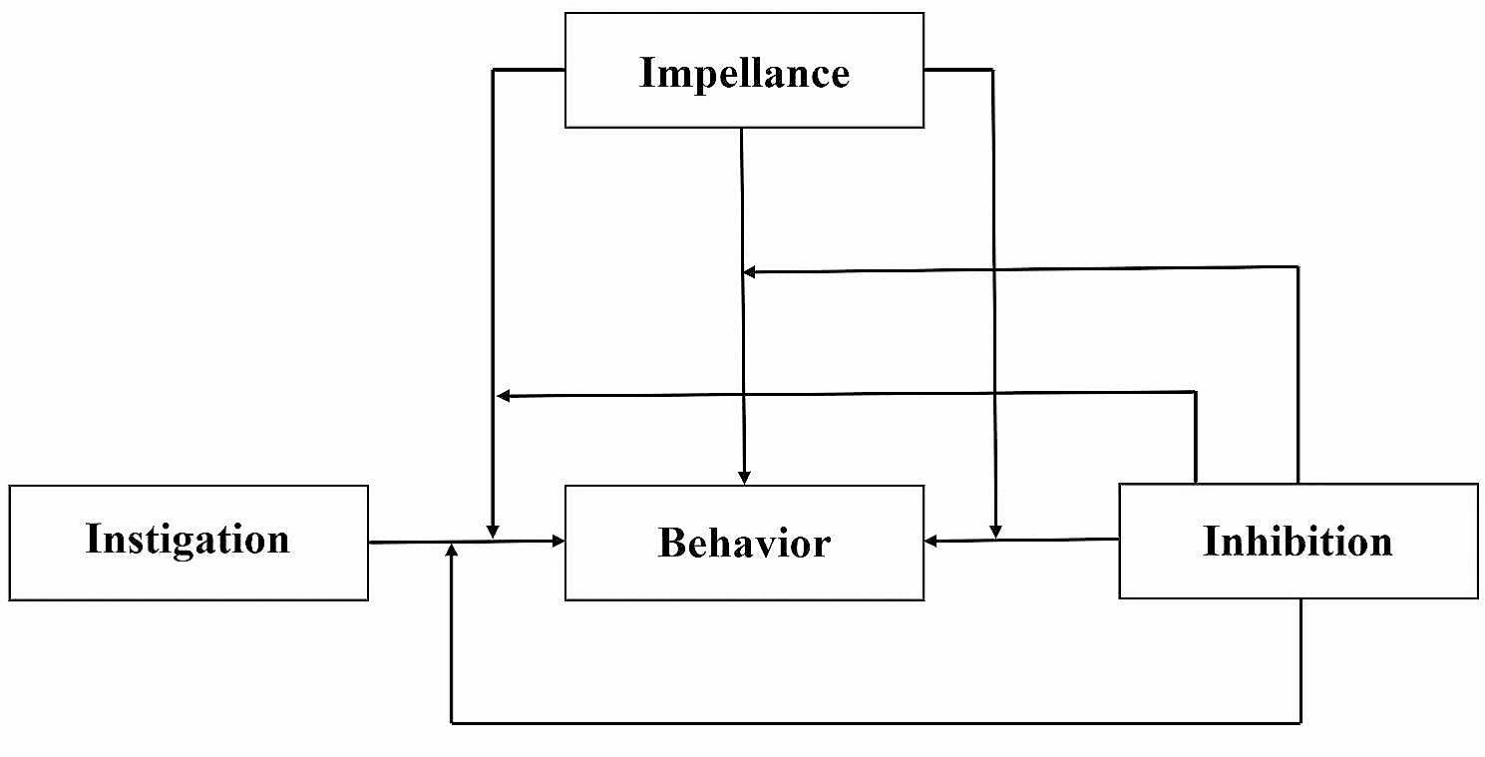
I^3^ structural model

The I^3^ model [19] (Fig. 1) can be used to explain the impact mechanism of malicious online trolling and has been verified in previous studies on malicious online trolling [20, 21]. The I^3^ model posits that aggressive behavior is the outcome of the interaction among three factors: Instigation, impellance, and inhibition [22]. Instigation is defined as environmental factors that may provoke aggressive tendencies, such as cyber victimization experiences, misbehavior provocation [23, 24]. Impellance is defined as the extent to which personal characteristics and environmental factors affect an individual’s propensity to attack, such as trait anger and online disinhibition [20, 25]. Inhibition is defined as individual and environmental factors that reduce or prevent the occurrence of aggressive behavior, such as self-control and trait mindfulness [20, 26]. Therefore, our proposal is to examine the impact of antisocial media exposure as an instigator of malicious online trolling behavior, whether hostile attribution bias (impellor) acted as a mediator and whether empathy (inhibitor) moderated it via a direct or indirect pathway.

### Antisocial media exposure and malicious online trolling

Antisocial media content refers to a range of unhealthy and norm-violating risky behaviors disseminated through online media, including violence, alcohol abuse, sexual harassment, and theft [27], among others. For example, users may encounter graphic violent videos or images while using social media, which are typical examples of anti-social media content. These are particularly popular among young people [28, 29]. According to social cognitive theory [30], individuals have the potential to acquire antisocial behaviors when exposed to media content portraying actors being supported or rewarded for engaging in such actions. This may increase their propensity for aggressive behavior. Some researchers have suggested a link between frequent exposure to glorified antisocial media and an elevated probability of engaging in aggressive behavior [31, 32]. Researchers have identified a significant correlation between antisocial media exposure and cyberbullying [14]. Furthermore, empirical research by den Hamer and Koniji [33] has found that antisocial media exposure can lead to cyberbullying. Malicious online trolling is often considered an abusive and aggressive behavior [11] that shares similar characteristics with cyberbullying. Therefore, regular contact with antisocial media may have a similar effect on malicious online trolling behavior. On the basis of the above, we inferred that antisocial media exposure is a key stimulus for malicious online trolling.

### Hostile attribution bias as a mediator

The General Aggression Model (GAM) proposed by Anderson and Bushman [34] is a comprehensive framework that explains and predicts aggressive behavior by considering individual traits, situational factors, and cognitive and affective processes. The GAM posits that situational factors increase an individual’s aggressive behavior through aggressive cognition. Hostile attribution bias refers to the inclination of individuals to interpret the words and actions of those around them as hostile when surrounding cues are ambiguous or unpredictable [35]. It is a typical form of aggressive cognition. Accordingly, our proposition suggested that hostile attribution bias serves as mediator between antisocial media exposure (a situational factor) and malicious online trolling (aggressive behavior). The Social Information Processing (SIP) model [36] posits that individuals would experience the process of hostile attribution bias prior to exhibiting aggressive behavior [37]. Specifically, when individuals interpret others’ intentions as hostile, this perception of hostility will prompt individuals to generate aggressive responses [38]. Research found that a high level of hostile attribution bias is a key factor in both the initiation and perpetuation of aggressive behavior [39]. Several longitudinal and empirical studies have examined the impact of hostile attribution bias on aggression [40, 41], and its positive relation with different forms of aggression [42]. Recently, researchers have examined the relation between hostile attribution bias and cyberbullying, revealing a positive correlation between the two [43–45]. Therefore, we proposed that a positive correlation between hostile attribution bias and malicious online trolling.

The script theory [46] suggests that individuals exposed to violent content through media learn corresponding aggressive scripts, and repeated exposure makes the pathways linking concepts and scripts easier to activate. Therefore, we believed that individuals who frequently watch antisocial media content are likely to form various situationally generalized hostile cognitive scripts. Once certain stimuli activate these hostile cognitive scripts, individuals will exhibit corresponding hostile cognition associated with the scripts. Many studies have demonstrated the connection between violent media content and hostile cognition [47–49]. A longitudinal study of more than 900 participants found that individuals’ levels of hostile cognitions increased over time after exposure to violent content [37]. In the new media era, antisocial content has a wider coverage than violent content, leading to greater malignant effects on individuals [50]. Malicious online trolling and cyberbullying share similar attack characteristics and both cause great harm to the victims [7]. Therefore, we speculated that there might be a positive correlation between antisocial media exposure and hostile attribution bias.

### Empathy as a moderator

The I^3^ model posits that inhibitors buffer the effects of instigators and impellors on aggressive behavior [22, 51, 52]. Empathy is commonly referred as an emotional response originating from another person and aligning with that person [53]. Hendry et al. [54] have used the I^3^ model in their research to confirm that empathy serves as an inhibitory factor for online antisocial behavior. We therefore further hypothesized that empathy may act as a moderator that reduces the direct as well as indirect effects of antisocial media exposure (instigator) on malicious online trolling (through the influence of hostile attribution bias as an impellor).

On the basis of the Differential Susceptibility to Media Effects Model (DSMM), specific individual factors serve as regulatory factors between exposure to risky content and behavior [55]. Individuals with higher levels of empathy may experience more emotional distress when exposed to antisocial media content, leading to a resistance to negative behaviors. Consequently, individuals with higher levels of empathy exhibit reduced tendency to participate in malicious online trolling in contrast to those demonstrating lower empathic levels. Mitchell et al. [56] have demonstrated that empathy mitigates the adverse impact of antisocial media content, such as sexual and violent content, on behavior. Therefore, we postulated that the correlation between antisocial media exposure and malicious online trolling would weaken when empathy is high. According to the revised model of SIP proposed by Lemerise and Arsenio [57], individuals with low levels of empathy have lower analytical abilities when extracting and encoding the same media content, which increases the likelihood of interpreting it as hostile in ambiguous and uncertain situations. Because individuals with low empathy feel less emotional distress [58], individuals with lower levels of empathy after exposure to antisocial media content may passively accept or actively seek out more antisocial content, and thus be more likely to develop hostile perceptions. On the basis of this, we speculated that empathy would reduce the impact of antisocial media exposure on hostile cognition.

In addition, even if individuals have higher levels of hostile cognition, those with high levels of empathy can, through perceiving and predicting others’ emotional states and maximizing the analysis of surrounding information, to some extent, restrain the negative impact brought by hostile cognition, avoid harmful behavior, and reduce harm to others [59]. Based on this, we speculated that the link between hostile attribution bias and malicious online trolling is attenuated among individuals when empathy is high.

### Current study

**Fig. 2:**
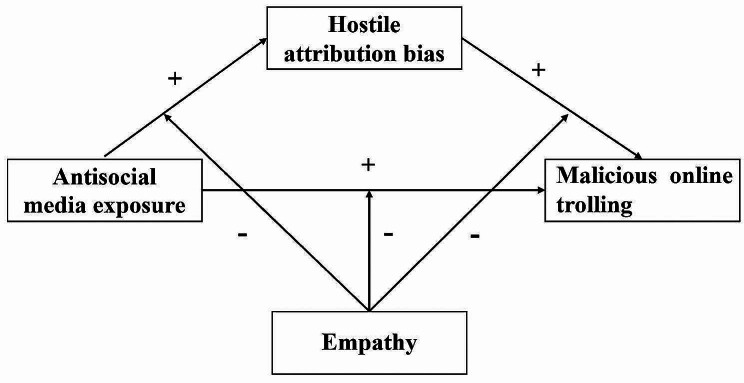
The proposed moderated mediation model

In this study, we broadened previous research by exploring the relationship between online environmental factors (antisocial media exposure) and malicious online trolling. Furthermore, for the first time, we combined the I^3^ model with the simultaneous inclusion of instigation (antisocial media exposure), impellance (hostile attribution bias), and inhibition (empathy) to explore their impact on malicious online trolling. In particular, we analyzed the function of hostile attribution bias as a mediator in antisocial media exposure and malicious online trolling, and empathy as a moderator. The current study established a moderated mediation hypothesis model (Fig. 2). We proposed three hypotheses:

#### H1

Antisocial media exposure is positively correlated with malicious online trolling.

#### H2

Hostile attribution bias mediates the relationship between antisocial media exposure and malicious online trolling. In particular, both antisocial media exposure and malicious online trolling are positively related to hostile attribution bias.

#### H3a

Empathy moderates the direct path between antisocial media exposure and malicious online trolling. Specifically, when individuals have higher levels of empathy, the effect of antisocial media exposure on malicious online trolling is weakened.

#### H3b

Empathy moderates the relationship between antisocial media exposure and hostile attribution bias. Specifically, when individuals have higher levels of empathy, the effect of antisocial media exposure on hostile attribution bias is weakened.

#### H3c

Empathy moderates the relationship between hostile attribution bias and malicious online trolling. Specifically, when the individual’s empathy level is high, the effect of hostile attribution bias on malicious online trolling is weakened.

## Method

### Participant and procedure

In this study, a questionnaire was administered to college students from several provinces in China (such as Sichuan and Guangdong) using convenience sampling, receiving 1322 responses. All participants reviewed the informed consent form and filled out the questionnaire anonymously. They read the neutral research instructions, which emphasized that there were no preset answers when the study was conducted and encouraged answers based on real experiences and feelings. Participants had the option to terminate the study at any time. No participant received compensation for participating in this study. Among them, 63 individuals (4.77%) either did not complete all the questions or did not fill out the questionnaire as required; their questionnaires were marked as invalid and deleted. The final valid questionnaires amounted to 1259 (*M*_*age*_ = 20.74, *SD* = 1.97), with 570 (45.27%) men and 689 (54.73%) women, giving a validity rate of 95.23%. The entire study procedures received approval from the ethical review board of the first author’s institution.

### Measures

#### Antisocial media exposure

The antisocial media exposure scale was used subscales of the Content-based Exposure Measure (C-EM) developed by den Hamer et al. [27]. In the current study, the scale was translated into Chinese and back-translated by two psychology professors, and modified in light of the current situation of Internet use in China. In previous studies, the scale has shown adequate validity and internal consistency in the Chinese context [14]. The scale comprises 8 items, utilizing a 1 to 5 rating (1 = never, 5 = always). We calculated the average score for each participant, with higher scores indicating a greater frequency of individual exposure to antisocial media content. We performed confirmatory factor analysis (CFA) with a fit index of χ^2^/ df = 3.325; TLI = 0.991; CFI = 0.996; SRMR = 0.011; RMSEA = 0.043, and a Cronbach’s α was 0.936.

#### Malicious online trolling

The study assessed malicious online trolling behavior using the Revised Global Assessment of Internet Trolling (GAIT-R) [60], which is an adaptation of the original Global Assessment of Internet Trolling (GAIT) scale developed by Buckels et al. [3]. The Chinese version was translated by Li et al. [61]. The scale comprises 8 items, utilizing a 1 to 5 rating (1 = strongly disagree, 5 = strongly agree), with higher total scores indicating more severe malicious online trolling behavior. In this research, the Cronbach’s α was 0.891.

#### Hostile attribution bias

Hostile attribution bias was evaluated using the Word Sentence Association Paradigm for Hostility (WSAP-Hostility) developed by Dillon et al. [62]. The Chinese version of the scale has also undergone a rigorous translation and back-translation process and has shown adequate validity and internal consistency in the Chinese context [63]. It has 16 contextually ambiguous sentences, each followed by an adjective related to hostility. Participants were assigned the task of evaluating the resemblance between provided sentences and hostility-related adjectives, utilizing a 1 to 6 rating (1 = not similar at all, 6 = completely similar). The average score for each participant was calculated, with a higher score indicating more severe hostile attribution bias. We performed confirmatory factor analysis (CFA) with a fit index of χ^2^ / df = 3.402; TLI = 0.992; CFI = 0.993; SRMR = 0.012; RMSEA = 0.032, and a Cronbach’s α was 0.933.

#### Empathy

Empathy was assessed using the Basic Empathy Scale developed by Jolliffe and Farrington [64], with a Chinese version revised by Li et al. [65]. It has 20 items, utilizing a 1 to 5 rating (1 = completely disagree, 5 = completely agree). A higher score indicated a stronger level of basic empathy. In this research, the Cronbach’s α was 0.696.

### Data analysis

Statistical analysis was conducted using SPSS 26.0, AMOS 24.0. First, we examined whether the data followed normal distribution. The skewness and kurtosis of antisocial media exposure, hostile attribution bias, empathy, and malicious online trolling all met the standard [66]. We used Harman’s single-factor test to evaluate common method bias. Based on the standard proposed by Kock et al. [67], if the first factor’s variance explained is less than 50%, it indicates that common method bias is unlikely to significantly affect the validity of the study results. The descriptive statistics and pearson correlation coefficients for the study variables are presented in Table 1. All variables were standardized before conducting the mediation and moderation analyses. Specifically, the mediation model was tested using the PROCESS macro model 4, and the moderation model was tested using PROCESS macro model 59 [68]. The 95% confidence intervals (CIs) for the mediating and moderating effects were calculated using the bias-corrected percentile bootstrap method (*N* = 5000). Statistical significance was attributed to these effects when the confidence interval did not include zero.

## Result

### Common method bias test

According to the results of Harman’s single-factor test that the first common factor contributed 12.88% of the variance (< 50%). This suggests that there was no significant common method bias in the research data.

### Preliminary analyses

Descriptive statistics and correlations were calculated for each variable (Table 1). The results indicated that antisocial media exposure and malicious online trolling were significantly positively correlated (*r* = 0.575, *p* < 0.001), supporting Hypothesis 1. Hostile attribution bias was positively correlated with antisocial media exposure and malicious online trolling, but negatively correlated with empathy (*r* = 0.452, 0.585, -0.355, *ps* < 0.001). Empathy was negatively correlated with malicious online trolling (*r* = -0.586, *p* < 0.001) and antisocial media exposure (*r* = -0.315, *p* < 0.001). In addition, gender was correlated with antisocial media exposure, hostile attribution bias, empathy, and malicious online trolling (*r* = -0.121, -0.158, 0.392, -0.382, *ps* < 0.001). Therefore, gender was considered as a covariate in subsequent analyses.

**Table 1:** Descriptive statistics and correlations among study variables

Variable	M	SD	Gender	AME	HAB	EM	MOT
Gender	-	-	-				
Antisocial media exposure (AME)	17.603	7.353	-0.121***	-			
Hostile attribution bias (HAB)	49.749	16.686	-0.158***	0.452***	-		
Empathy (EM)	66.518	8.584	0.392***	-0.315***	-0.353***	-	
Malicious online trolling (MOT)	14.587	7.439	-0.382***	0.575***	0.585***	-0.586***	-

*NoteM* = mean; *SD* = Standard Deviation; Gender was dummy coded such that 0 = men and 1 = women. ***p < 0.001

### Testing for the mediation effect

The mediating effects of hostile attribution bias were investigated using Hayes’ PROCESS macro (Model 4), with gender serving as a covariate. The results of the mediation effect analyses are presented in Table 2, with antisocial media exposure positively predicting hostile attribution bias (*b* = 0.439, *p* < 0.001), as well as hostile attribution bias positively predicting malicious online trolling (*b* = 0.373, *p* < 0.001). In the presence of hostile attribution bias, antisocial media exposure continued to positively predict malicious online trolling (*b* = 0.373, *p* < 0.001, 95%CI [0.331, 0.415]). In addition, the mediating effect of hostile attribution bias was significant (*b* = 0.164, *p* < 0.001, 95%CI [0.134, 0.195]). In sum, hostile attribution bias partially mediated the relationship between antisocial media exposure and malicious online trolling, validating hypothesis 2.

**Table 2:** Testing the mediating effect of hostile attribution bias

	Hostile attribution bias (Model 1)			Malicious online trolling (Model 2)		
	*b*	*SE*	*t*	*b*	*SE*	*t*
Gender	-0.211	0.051	-4.166***	-0.559	0.039	-14.320***
Antisocial media exposure	0.439	0.025	17.431***	0.373	0.022	17.334***
Hostile attribution bias				0.373	0.022	17.237***
*R* ^*2*^	0.215			0.539		
F	171.860***			489.171***		

*Note* ***p < 0.001

### Testing for the moderated mediation

**Fig. 3:**
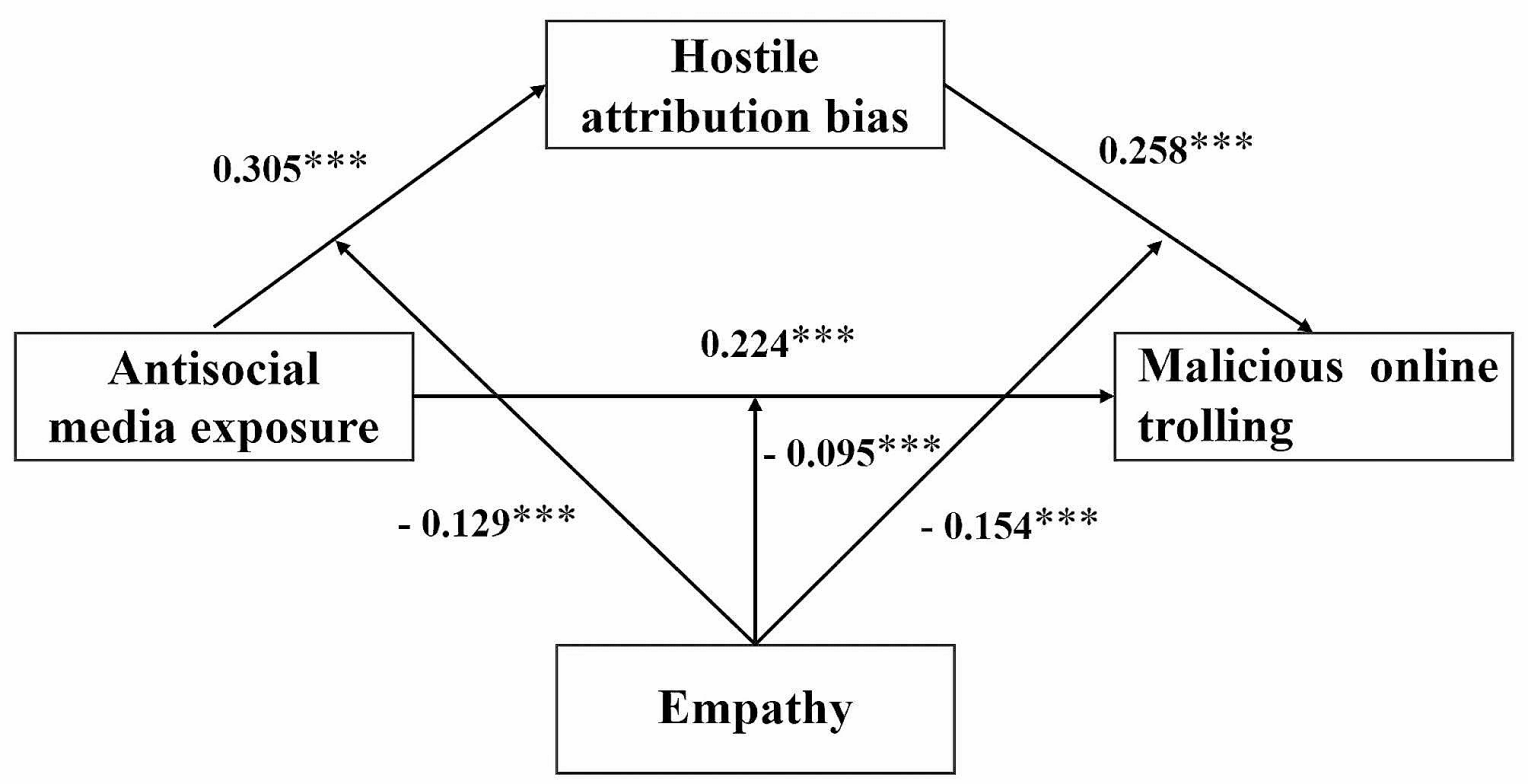
Path coefficients of the moderated mediation model

**Fig. 4:**
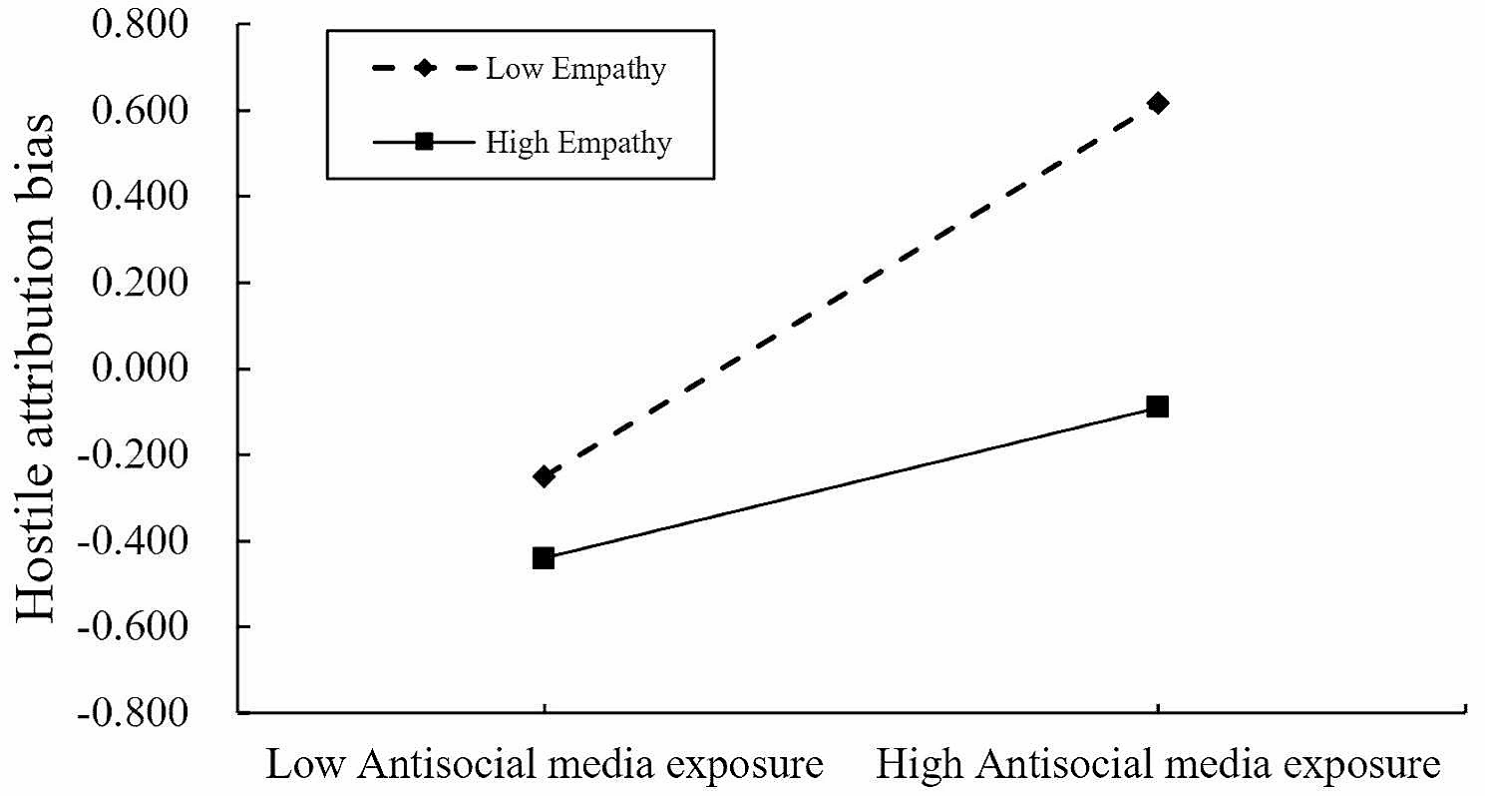
Interaction between antisocial media exposure and empathy on hostile attribution bias

**Fig. 5:**
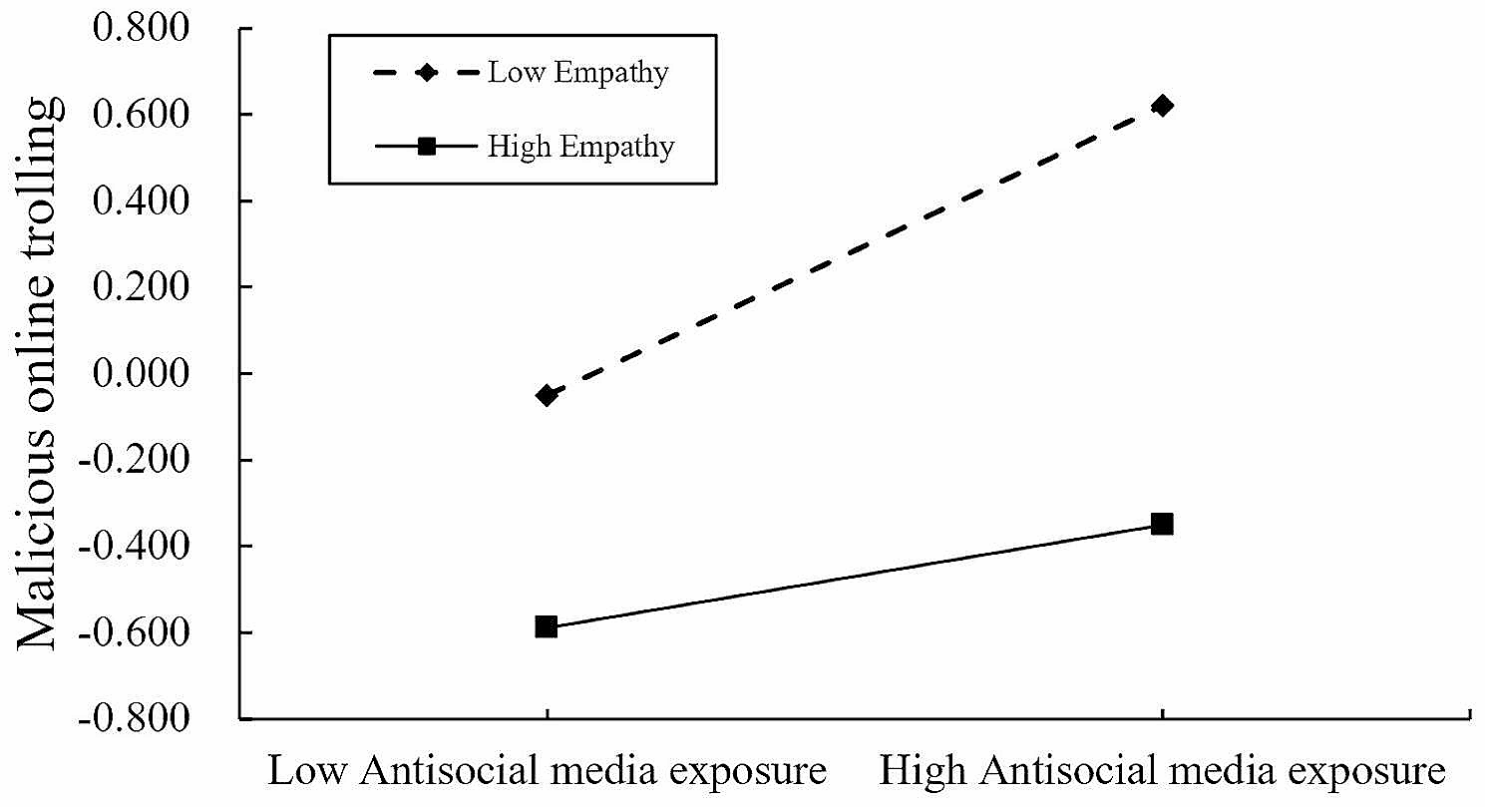
Interaction between antisocial media exposure and empathy on malicious online trolling

**Fig. 6:**
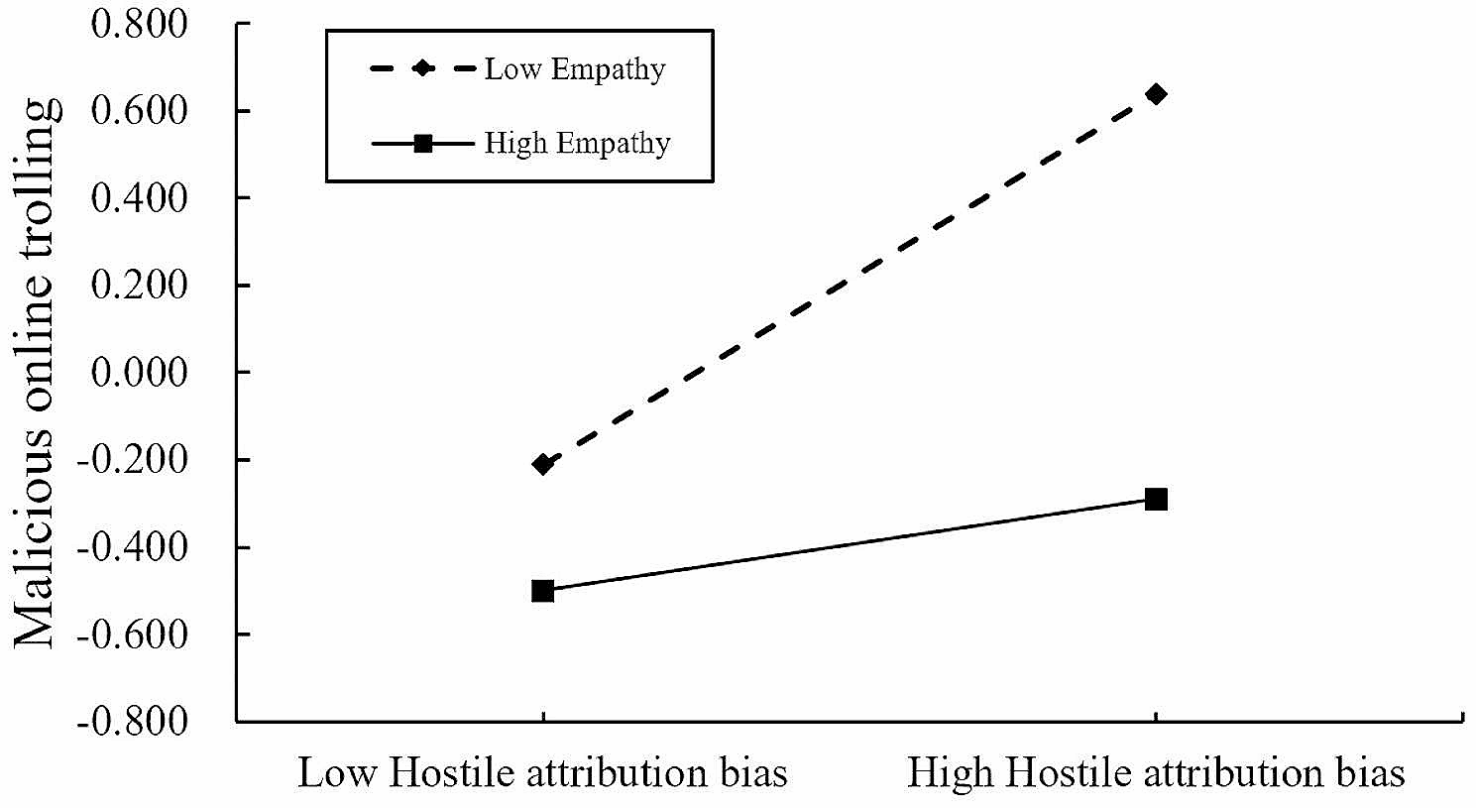
Interaction between hostile attribution bias and empathy on malicious online trolling

The moderating effects of empathy were investigated using Hayes’ PROCESS macro (Model 59), with gender serving as a covariate. As shown in Table 3; Fig. 3, the interaction terms of antisocial media exposure and empathy both significantly and negatively predicted malicious online trolling as well as hostile attribution bias (*b* = -0.095, *p* < 0.001, 95% CI [-0.129, -0.062]; *b* = -0.129, *p* < 0.001, 95% CI [-0.173, -0.084]), suggesting that empathy moderated the direct link between antisocial media exposure and malicious online trolling as well as the link between antisocial media exposure and hostile attribution bias. Meanwhile, the interaction term between empathy and hostile attribution bias significantly negatively predicted malicious online trolling (*b* = -0.154, *p* < 0.001, 95% CI [-0.188, -0.121]), suggesting that the relationship between hostile attribution bias and malicious online trolling was also moderated by empathy. To elaborate on the moderating effects of the three pathways of empathy, we conducted a simple slope test. The results indicated that, as shown in Fig. 4, antisocial media exposure had a significantly positive predictive effect on hostile attribution bias, and this relationship was moderated by the level of empathy. Simple slopes indicated that under high and low levels of empathy, antisocial media exposure positively predicted hostile attribution bias, but compared to low levels of empathy (*b*_simple_ = 0.434, *t* = 15.934, *p* < 0.001), the predictive effect is weaker under high empathy levels (*b*_simple_ = 0.176, t = 4.046, *p* < 0.001). As shown in Fig. 5, antisocial media exposure positively predicted malicious online trolling of individuals at different levels of empathy, but compared to individuals with low empathy (*b*_simple_ = 0.319, *t* = 14.313, *p* < 0.001), the predictive effect was weakened for individuals with high empathy (*b*_simple_ = 0.128, *t* = 4.219, *p* < 0.001). Similarly, as shown in Fig. 6, hostile attribution bias positively predicts malicious online trolling of individuals at both high and low empathy levels, but compared to individuals with low empathy (*b*_simple_ = 0.413, *t* = 16.172, *p* < 0.001), the predictive effect was weaker under high empathy levels (*b*_simple_ = 0.104, *t* = 3.847, *p* < 0.001). Therefore, hypothesis 3 was supported.

**Table 3:** Testing the moderating effect of empathy

	Hostile attribution bias (Model 1)			Malicious online trolling (Model 2)		
	*b*	*SE*	*t*	*b*	*SE*	*t*
Gender	-0.031	0.053	-0.579	-0.340	0.036	-9.398***
Antisocial media exposure	0.305	0.028	10.748***	0.224	0.021	10.896***
Hostile attribution bias				0.258	0.019	13.272***
Empathy				-0.294	0.019	-15.151***
Int-1	-0.129	0.023	-5.678***			
Int-2				-0.095	0.017	-5.591***
Int-3				-0.154	0.017	-9.023***
*R* ^2^	0.273			0.658		
F	117.476***			400.688***		

*Notes* Int-1 = Antisocial media exposure × Empathy (to Hostile attribution bias); Int-2 = Antisocial media exposure × Empathy (to Malicious online trolling); Int-3 = Hostile attribution bias × Empathy (to Malicious online trolling). ***p < 0.001

## Discussion

This study examined the association and potential mechanisms between antisocial media exposure and malicious online trolling. Findings showed that antisocial media exposure and malicious online trolling were positively related, with hostile attribution bias acting as a mediator. In addition, empathy moderated the relations between hostile attribution bias and malicious online trolling, antisocial media exposure and hostile attribution bias, and the direct path from antisocial media exposure to malicious online trolling. This study identified a key risk factor for malicious online trolling among Chinese college students expanded understanding of relevant potential mechanisms and contributed to understanding how to mitigate this impact.

### The relationship between antisocial media exposure and malicious online trolling

The hypothesis that antisocial media exposure among college students was positively associated with malicious online trolling was supported in our study. Individuals exposed to antisocial media may engage in malicious online trolling due to observational learning and self-reinforcement, which is roughly consistent with social cognitive theory [30] and previous empirical research has indicated that antisocial media exposure can predict cyber aggression [33]. The research results were consistent with the I^3^ model, suggesting that antisocial media exposure may be an instigation factor for malicious online trolling [22, 69]. The quantity of antisocial media content continues to increase, and its negative impact on individuals is stronger than that of singular violent content [14]. Long-term exposure increases the frequency of aggression in individuals [32]. To release their aggression, individuals may select malicious online trolling as a low-risk outlet, which does not require specific targets or motives [70, 71], thereby increasing the likelihood of engaging in such behavior.

### The mediating effect of hostile attribution bias

The results of this study showed that hostile attribution bias played a mediating role between antisocial media exposure and malicious online trolling. Although this study utilized a cross-sectional design and could not directly infer a causal relationship, the result indicated that hostile attribution bias may have played a role in cognitive processes, and antisocial media exposure might have affected malicious online trolling through this cognitive pathway. It was also consistent with previous research findings that hostile attribution cognition could mediate the relationship between negative media content and cyber-aggression [45, 48]. Hostile attribution bias was a negative consequence of antisocial media exposure. The more exposure to antisocial media, the more likely individuals are to interpret uncertain environments with hostility [72], which aligns with script theory. Hostile attribution bias can trigger anger rumination, depleting cognitive resources for inhibiting aggression [73]. When individuals interpret the current online situation as hostile, they believe they have a reason to be angry, and therefore, to vent their frustration, they are likely to make aggressive responses [38], thereby increasing malicious online trolling. Additionally, these results also supported the I^3^ model [69]. Massa et al. [74] has suggested that hostile cognition may be a driving force leading to aggressive responses. In this study, hostile attribution bias served as an impellance factor promoting malicious online trolling behavior.

### The moderating effect of empathy

As expected, empathy, directly and indirectly, regulated the relationship between antisocial media exposure and malicious online trolling via hostile attribution bias. Previous empirical research has demonstrated that empathy serves as a significant moderator of online antisocial behavior [56], but our study revealed for the first time that empathy acted as a moderator in the connection between antisocial media exposure and malicious online trolling via hostile attribution bias. The result that empathy as a protective factor attenuates the effects of antisocial media exposure on malicious online trolling is consistent with the Differential Susceptibility to Media Effects Model [55]. Individuals with higher levels of empathy exhibit a reduced inclination to engage in malicious online trolling after exposure to antisocial media content; these individuals experience higher levels of distress and lower levels of pleasure when viewing antisocial media content [75]. Thus, as opposed to obtaining pleasure through malicious online trolling [8], individuals with high empathy are inclined to seek relief from emotional stress, thus reducing the likelihood of committing malicious online trolling behaviors. Meanwhile, the effect of antisocial media exposure on hostile cognitive bias is moderated by empathy. When exposed to the same content, individuals with low empathy have weaker abilities to understand and interpret ambiguous content compared to those with high empathy [76]. Consequently, they have a propensity to develop hostile cognitive bias. Additionally, as empathy levels increase, the positive prediction of hostile attribution bias on malicious online trolling is attenuated. Individuals with high empathy can better understand the meaning that others intend to express in ambiguous situational clues, restrain the negative effects of hostile cognition [59], predict and understand others’ emotions, and do not derive pleasure from engaging in malicious online trolling behavior that causes pain to others.

### Limitations and implications

The study could benefit from improvements in several areas. First, the current study is a cross-sectional study and cannot infer causal relationships. The research data were based solely on self-reports. Although participants were informed that the survey was completely anonymous, potential bias issues may still exist. To enhance the clarity of the study structure, it may be useful to conduct experimental manipulations to examine the impact of antisocial media content and empathy on malicious online trolling. This study explored malicious online trolling behavior among college students. However, it is possible that other groups, such as high school students or working populations, may also engage in malicious online trolling. Future research could broaden the scope to include these groups. Additionally, this study selected individual protective factors (empathy) to investigate their positive impact on reducing malicious online trolling. However, other factors, such as mindfulness, may also have a mitigating effect on malicious online trolling. Future research can focus on exploring similar variables.

Despite some limitations, this study has certain theoretical and practical significance. First, this study expands the scope of application of the I^3^ model content, reveals that the risky media content that people are exposed to on a daily basis is a stimulus for the generation of online trolling behaviors, and finds that empathy plays a key role in inhibiting online trolling behaviors, which provides new ideas for the future implementation of online trolling behaviors. In addition, this study, from the perspective of macro-network environmental factors, deeply explores how these factors affect online trolling behaviors and their mechanisms of action, providing new ideas for subsequent research. Based on the results of this study, it is recommended that policymakers and social platform operators jointly assume regulatory responsibilities and formulate clear review policies based on local laws and cultural backgrounds to ensure the rapid identification and deletion of malicious online content. At the same time, professional capabilities should be improved, and the ability to identify antisocial media content should be improved in combination with current artificial intelligence technology to intercept illegal content and prevent it from being widely disseminated. In addition, we recommend that families, schools, and communities work together. Even if some schools have limited psychological teaching resources, relevant educational courses can still be carried out to enhance students’ ability to identify malicious online trolling content, improve media literacy, and cultivate comprehensive personality development. In addition, being exposed to beautiful and positive media content (such as helping others and green nature) can improve empathy levels [77, 78], promote the improvement of healthy personality, and reduce online content from the source.

## Conclusion

Based on the I^3^ model, this study reveals the mediating role of hostile attribution bias between antisocial media exposure and online trolling. In addition, empathy has a significant impact on both the direct and indirect links between antisocial media exposure and online trolling. Therefore, the prevention and intervention strategies for online trolling should comprehensively consider external influencing factors (e.g., antisocial media exposure) and individual internal factors (e.g., hostile attribution bias and empathy).

## Data Availability

The dataset will be available from the corresponding author on reasonable request.
